# Detection of influenza A virus in aerosols of vaccinated and non-vaccinated pigs in a warm environment

**DOI:** 10.1371/journal.pone.0197600

**Published:** 2018-05-21

**Authors:** Victor Neira, Matt Allerson, Cesar Corzo, Marie Culhane, Aaron Rendahl, Montserrat Torremorell

**Affiliations:** 1 Departamento de Medicina Preventiva, Facultad de Ciencias Veterinarias y Pecuarias, Universidad de Chile, La Pintana, Santiago, Chile; 2 Department of Veterinary Population Medicine, College of Veterinary Medicine, University of Minnesota, St. Paul, Minnesota, United States of America; University of South Dakota, UNITED STATES

## Abstract

The 2009 influenza pandemic, the variant H3N2v viruses in agricultural fairs and the zoonotic poultry H5N9 infections in China have highlighted the constant threat that influenza A viruses (IAV) present to people and animals. In this study we evaluated the effect of IAV vaccination on aerosol shedding in pigs housed in warm environmental conditions. Thirty-six, three-week old weaned pigs were obtained from an IAV negative herd and were randomly allocated to one of 4 groups: 1) a homologous vaccine group, 2) a heterologous multivalent vaccine group, 3) a heterologous monovalent group and, 4) a non-vaccinated group. After vaccination pigs were challenged with the triple reassortant A/Sw/IA/00239/04 H1N1 virus. Environmental temperature and relative humidity were recorded throughout the study. Nasal swabs, oral fluids and air samples were collected daily. All samples were tested by RRT-PCR and virus isolation was attempted on positive samples. Average temperature and relative humidity throughout the study were 27°C (80°F) and 53%, respectively. A significantly higher proportion of infected pigs was detected in the non-vaccinated than in the vaccinated group. Lower levels of nasal virus shedding were found in vaccinated groups compared to non-vaccinated group and IAV was not detected in air samples of any of the vaccinated groups. In contrast, positive air samples were detected in the non-vaccinated group at 1, 2 and 3 days post infection although the overall levels were considered low most likely due to the elevated environmental temperature. In conclusion, both the decrease in shedding and the increase in environmental temperature may have contributed to the inability to detect airborne IAV in vaccinated pigs.

## Introduction

Influenza A virus (IAV) is a negative sense single stranded RNA virus belonging to the *Orthomyxoviridae* family. IAV affects people and many animal species including pigs. In pigs IAV is a primary cause of respiratory disease which is characterized by anorexia, fever, sneezing, coughing, rhinorrhea and lethargy [1–3].

Pigs play an important role in the ecology of IAV infections. Pigs can serve as mixing vessels resulting in the generation of novel IAVs [4], but also serve as reservoirs for strains of zoonotic and pandemic potential [5–7]. IAV is endemic in swine and distributed worldwide [8–10]. The 2009 H1N1 pandemic and the H3N2 variant events in agricultural fairs have highlighted the risk of IAV transmission from pigs to humans and vice-versa making understanding transmission routes a priority [11–13].

IAV can be transmitted by direct and indirect contact and aerosols [14]. In swine, there is increased evidence of the role that aerosols play in IAV transmission and IAV has been detected and isolated from aerosols generated from pigs with and without immunity under both experimental [15–17] and field conditions [18,19]. Epidemiological studies have found an association between seropositivity and pig farm density [20,21] and a relationship between the proximity of pig farms to turkey flocks and the likelihood of turkey flock seropositivity to swine-origin IAV [22] has been described. Overall these observations highlight the importance of IAV aerosols on transmission and geographic dissemination.

Influenza vaccination is used in swine populations as a strategy to mitigate clinical effects and the economic impact of IAV infections [23,24]. Experimentally, vaccines have proved to reduce lung lesions, shedding [9,17,25,26] and transmission rates in pigs; however, this depends on the level of cross-protection between the vaccine and challenge strains and whether immunity is active or passive [27–29].

Experimental transmission studies performed with ferrets have reported that IAV transmission is depent on temperature and relative humidity [30] and that high temperatures (30°C) may decrease the risk of airborne transmission [31,32]. This environmental effect may explain why human seasonal influenza peaks in midwinter under dry and cold conditions [32,33]. However, in swine farms although peaks of IAV infection can also seasonal [34] farms remain endemically infected throughout the year [8] and there is still a need to understand the main routes of IAV transmission.

Overall, IAV vaccination is one of the few available control tools in pigs but the effect of vaccination on the generation of aerosols is largely unknown; therefore, the objective of this study was to assess the effect of IAV vaccination on the generation of IAV bioaerosols under warm conditions in pigs with varying degrees of cross-protective immunity. Data regarding vaccination and its potential impact on aerosol generation may provide fundamental information for control programs in areas where aerosol transmission is considered significant.

## Materials and methods

### Experimental design

#### Animals and vaccines

Thirty-six, three-week old pigs were purchased from an IAV seronegative herd based on testing using a commercially available nucleoprotein (NP) ELISA test [35]. Pigs were randomly assigned to one of four experimental groups: a) group 1 (VAC-HOM), pigs were vaccinated with a homologous vaccine (Newport Labs, Worthington, MN) to the A/Sw/IA/00239/04 (H1N1 beta clade 1A.2) challenge virus; b) group 2 (VAC-HET MULTI) pigs were vaccinated with a commercial heterologous multivalent vaccine, containing A/Sw/NC/031/05 (H1N1 delta2 clade 1B.2.1), A/Sw/MO/069/05 (H3N2 Cluster IV) and A/Sw/IA/110600/00 (H1N1 gamma2 clade 1A.3.2) IAV strains (FluSure XP^®^, Zoetis, New York, NY); c) group 3 (VAC-HET MONO) pigs were vaccinated with a commercial heterologous monovalent vaccine, containing A/CA/04/2009 (H1N1 npdm clade 1A.3.3.2) (Flusure^®^ Pandemic, Zoetis, New York, NY) and d) group 4 (NON-VAC) pigs were vaccinated with a sterile phosphate saline solution (PBS) to serve as the unvaccinated control group. The four groups included represent 4 levels of protection against the challenge strain: homologous (group 1), heterologous (groups 2 and 3) and no protection (group 4). Pigs in all groups were vaccinated with 2 ml of vaccine by intramuscular injection with a 2-inch needle into the right neck muscles just caudal to the base of the ear and revaccinated 2 weeks after the first vaccination with their respective treatment products. The commercial vaccines were considered heterologous to the challenge virus based on their clade designations [36] and hemagglutinin (HA) gene sequences (sequence homology <95% between strains).

Animals were housed at the University of Minnesota College of Veterinary Medicine animal BSL-2 research facilities. Each group of animals was maintained in an separate isolation room, which had a dedicated air intake and solid floor area of 7.28 m^2^. Pigs were feed with a standard corn soy diet for nursery age pigs. Fresh water and feed were provided *at libitum*. Pens were cleaned twice per day and pigs were observed 3 times per day by the researchers to monitor clinical signs of IAV.

#### Experimental pig infection

Pigs were challenged with A/Sw/IA/00239/04 H1N1 two weeks after the second vaccination. This challenge strain was isolated from an outbreak of respiratory disease in pigs submitted to the University of Minnesota Veterinary Diagnostic Laboratory and has been previously used in experimental transmission studies that included detection of shedding and detection of IAV in aerosols [27,28]. Pigs were challenged intratracheally and intranasally with 1 ml of inoculum containing 1 x 10^6^ tissue culture infective dose (TCID_50)_/ml). Pigs were sedated by an intramuscular injection of Telazol^®^ (Fort Dodge Animal Health, Fort Dodge, Iowa, USA) at dose of 6mg/kg. Pigs were euthanized at 14 days post inoculation (dpi) with an overdose of pentobarbital 100 mg/kg (Fatal-Plus Solution^®^, 250 mL, Vortech Pharmaceuticals, Dearborn, MI, USA). Animals were cared for according to the University of Minnesota Institutional Animal Care and Use Committee (IACUC) and the IACUC committee approved this study (protocol # 1110A05802).

### Sampling and diagnostics assays

#### Animal sampling

Blood samples were collected via jugular venipuncture prior to the first vaccination, prior to challenge and on the day of euthanasia. Samples were then centrifuged at 2,500 RPM for 10 minutes to obtain sera. Sera were tested to detect IAV antibodies by hemagglutination inhibition (HI) assay and ELISA (see below). Nasal swabs and oral fluids were collected daily between 0 to 8 dpi (days post inoculation) and tested for IAV through real-time reverse transcription (RRT-PCR). Individual nasal swabs were obtained using rayon swabs containing Stuart broth media (BD BBLTM CultureSwabTM, Sparks, MD, USA). After collection, each swab was resuspended into 1.8 ml of minimum essential media (MEM) with 2% of bovine serum albumin (BSA), trypsin tosyl chlorophenyl ketone (TPCK) and antibiotics, and stored at -80°C until testing. Three oral fluid samples were collected per day per group at 8h intervals, using a cotton rope [37]. Oral fluids were centrifuged for 20 minutes at 5,000 RPM to recover the supernatant and were preserved at -80°C until testing.

#### Air sampling

Three air samples per day were collected from each group at 8 h intervals starting at 0 dpi using a cyclonic collector (Midwest Microtek, Brookings, South Dakota, USA). The air collector was suspended in the room 70 cm away from the wall and 80 cm above the floor. The pigs did not have direct contact with the collector. According to manufacturer specifications, the collector had a capacity of capturing 400L of air per minute. Ten ml of MEM with 2% BSA, trypsin TPCK and antibiotics were poured into the collector collection vessel. The collector was allowed to run for a period of 30 minutes. After sampling, a sterile syringe (Tyco-Healthcare, Kendall Monoject) was used to recover the fluid from the collection vessel, transferred to a 10 ml plastic sterile tube and stored at -80°C until testing.

A room-specific air collector was used to avoid cross contamination between treatments. Between every air sampling, the cyclonic collector was cleaned and disinfected with alkyl dimethly benzyl ammonium chloride (Lysol, Reckitt Benckiser, Wayne, NJ, USA) and after that it was swabbed and tested to assure proper cleaning and disinfection procedures.

#### Diagnostic assays

Hemagglutination inhibition (HI) assay was performed using the challenge virus A/Sw/IA/00239/04 H1N1 following a standard protocol [38]. HI titer ≥ 40 was considered postitive against the test virus. ELISA was performed using a commercial kit, Swine Influenza Virus Ab Test (IDEXX Lab, Westbrook, ME). This test detects serum antibodies against IAV nucleoprotein (NP) in swine [35]. ELISA results with a sample to negative (S/N) ratio < 0.673 were considered positive. Nasal and oral fluid samples were tested by real-time reverse transcription (RRT-PCR) to detect IAV matrix gene [39] and quantified using a control virus solution diluted in 10 fold serial dilutions and expressed as log 10 TCID_50_/ml equivalents. Virus isolation was attempted from air positive samples in Madin-Darby canine kidney (MDCK) cells. All diagnostic assays were performed at the University of Minnesota Veterinary Diagnostic Laboratory.

### Environmental measurements

Temperature (T°) and relative humidity (RH%) readings were obtained every 5 minutes throughout the study using automated loggers (ThermaData Temperature & Humidity Logger (Model #296–061 (HTD), ThermalWorks, Lindon, UT, USA). The logger was suspended in the room 10 cm away from the walls and 130 cm above the floor.

### Statistical methods

Differences in HI (log2 transformed) and ELISA antibody levels pre and post-vaccination were analyzed by ANOVA. Kaplan Meier survival analysis was performed between groups to compare cumulative percentages of infected pigs per day. In order to determine differences in viral load shed in nasal swabs, Wilcoxon test was performed daily, correcting for multiple comparisons within each day with the Bonferroni-Holm correction. Temperature and RH% were analyzed by ANOVA. Statistical analyses were performed using STATA/SE 10.0 (StataCorp, College Station, TX, USA).

## Results

Pigs were both RRT-PCR and ELISA negative on arrival at the isolation units (results not shown) and RRT-PCR negative prior to inoculation. Table 1 summarizes the HI antibody levels against the challenge virus, the ELISA values prior to infection, and at necropsy. Prior to infection, pigs in the VAC-HOM group had significantly higher (p <0.05) HI titers compared to the other three groups. In the VAC-HET MONO group, 4 pigs had HI titers against the challenge strain, while pigs in the VAC-HET MULTI and the NON-VAC group were negative against the challenge strain. Pigs in the VAC-HET MULTI group were further tested by HI against the IAV strains contained in the commercial multivalent vaccine and tested positive (S1 Table). All groups were HI positive against the challenge strain at necropsy, however HI titers were statistically different between groups (p <0.05). ELISA results were in agreement with the HI titers.

**Table 1:** Hemagglutination inhibition (HI) geometric means against the influenza A virus challenge virus and ELISA titers from vaccinated and non-vaccinated pigs before inoculation and at necropsy.

	HI				ELISA			
	0 dpi^1^		Necropsy		0 dpi		Necropsy	
Group	N Pos^2^	Mean	N Pos	Mean	N Pos	Mean ± SD	N Pos	Mean ± SD
VAC-HOM	9/9	217^a^	9/9	691^a^	9/9	0.3 ± 0.08^a^	9/9	0.2 ± 0.07^a^
VAC-HET MULTI	0/9	20^b^	9/9	320^ab^	1/9	0.9 ± 0.13^b^	9/9	0.2 ± 0.07^a^
VAC-HET MONO	4/9	27.2^b^	9/9	508^a^	6/9	0.6 ± 0.23^c^	9/9	0.16 ± 0.04^a^
NON-VAC	0/9	10^b^	9/9	160^b^	0/9	0.9 ± 0.05^d^	8/9	0.49 ± 0.15^b^

VAC-HOM, homologous vaccine; VAC-HET MULTI, heterologous multivalent vaccine; VAC-HET MONO, heterologous monovalent vaccine; NON-VAC, non-vaccinated.

^1^ Days post inoculation

^2^ Number of positive pigs/total pigs

Letter superscripts show statistically significant differences between groups (p<0.05).

Fifteen thousand T° and RH% reads were recorded during the study period. Mean temperature and RH throughout the study was 27°C (80°F) (Min 18.4°C—Max 33.6°C) and 53% (Min 36.6%—Max 97.8%), respectively. Ninety percent of the readings were 53% ± 6.4 RH and 27 °C ±1.5 (S1 Fig). There were no differences between temperature and relative humidity among groups (Table 2).

**Table 2:** Mean and standard deviation readings of temperature (C) and relative humidity (%) obtained with automatic loggers throughout the study.

	VAC-HOM	VAC-HET MULTI	VAC-HET MONO	NON-VAC
	Mean ± SD	Mean ± SD	Mean ± SD	Mean ± SD
Temperature (C)	27.9 ± 1.7	28.2 ± 1.6	28 ± 1.7	27.5 ± 1.8
Relative humidity (%)	52.8 ± 8.7	53.6 ± 8.6	53.4 ± 9.6	53.5 ± 9.1

VAC-HOM: homologous vaccine; VAC-HET MULTI: heterologous multivalent vaccine; VAC-HET MONO: heterologous monovalent vaccine; NON-VAC: non-vaccinated.

Table 3 summarizes the RRT-PCR results and average daily viral load in nasal swabs, oral fluids and air samples. All groups had pigs with clinical signs of mild respiratory disease compatible with influenza characterized by watery nose, depression, sneezing and some coughing. No unexpected mortality was observed prior to the study endpoint. Pigs in the NON-VAC group became infected at 1 dpi and all pigs were positive by RRT-PCR. Pigs shed virus for 4 to 7 days. In the VAC-HET MULTI group, 8 out 9 pigs tested positive and shedding was observed for 1 to 5 consecutive days. In the VAC-HET MONO group 5 out 9 pigs were positive and shedding was also observed for 1 to 5 consecutive days. In contrast in the VAC-HOM group only one pig tested positive and was positive only one day (S1 Table).

**Table 3:** Number of RRT-PCR influenza A virus positive samples and average daily viral load (expressed in Log TCID50 equi/ml) in positive nasal swabs, oral fluids and air samples from vaccinated and non-vaccinated groups.

	VAC-HOM Positives/Total^2^ (Viral Load^3^)			VAC-HET MULTI Positives/Total (Viral Load)			VAC-HET MONO Positives/Total (Viral Load)			NON-VAC Positives/Total (Viral Load)		
DPI^1^	Nasal	Air	Oral Fluid	Nasal	Air	Oral Fluid	Nasal	Air	Oral Fluid	Nasal	Air	Oral Fluid
**0**	0/9 (0)	0/0 (0)	0/0 (0)	0/9 (0)	0/0 (0)	0/0 (0)	0/9 (0)	0/0 (0)	0/0 (0)	0/9 (0)	0/0 (0)	0/0 (0)
**1**	0/9 (0)	0/3 (0)	0/3 (0)	1/9 (1.5)	0/3 (0)	1/3 (1.7)	0/9 (0)	0/3 (0)	2/2 (3.2)	8/9 (2.5)	1/3 (0.3)	1/1 (1.2)
**2**	0/9 (0)	0/3 (0)	0/3 (0)	6/9 (1.9)	0/3 (0)	3/3 (3.4)	2/9 (2.2)	0/3 (0)	3/3 (1.8)	7/9 (3.0)	1/3 (1.3)	3/3 (3.4)
**3**	0/9 (0)	0/3 (0)	1/3 (2.9)	8/9 (2.2)	0/3 (0)	3/3 (2.9)	1/9 (2.4)	0/3 (0)	3/3 (2.7)	9/9 (3.6)	1/3 (1.0)	3/3 (2.9)
**4**	0/9 (0)	0/3 (0)	0/3 (0)	7/9 (2.7)	0/3 (0)	3/3 (4.0)	2/9 (1.9)	0/3 (0)	3/3 (2.7)	8/9 (3.9)	0/3 (0)	3/3 (3.8)
**5**	1/9 (1.6)	0/3 (0)	1/3 (1.2)	7/9 (3.2)	0/3 (0)	3/3 (3.2)	5/9 (2.6)	0/3 (0)	3/3 (2.7)	9/9 (3.1)	0/3 (0)	3/3 (3.6)
**6**	0/9 (0)	0/3 (0)	0/3 (0)	4/9 (2.0)	0/3 (0)	1/3 (2.6)	3/9 (2.5)	0/3 (0)	0/3 (0)	6/9 (2.3)	0/3 (0)	3/3 (2.9)
**7**	0/9 (0)	0/3 (0)	0/3 (0)	1/9 (1.3)	0/3 (0)	0/3 (0)	0/9 (0)	0/3 (0)	0/3 (0)	2/9 (2.4)	0/3 (0)	2/3 (2.5)
**8**	0/9 (0)	0/3 (0)	0/3 (0)	0/9 (0)	0/3 (0)	0/3 (0)	0/9 (0)	0/3 (0)	0/3 (0)	1/9 (1.1)	0/3 (0)	0/3 (0)

VAC-HOM: homologous vaccine; VAC-HET MULTI: heterologous multivalent vaccine; VAC-HET MONO: heterologous monovalent vaccine; NON-VAC: non-vaccinated.

^1^ Days post-inoculation

^2^ Number of positive samples / total number of samples

^3^ Average daily viral load in positive pigs expressed in Log TCID_50_ equivalents/ml.

**Fig 1:**
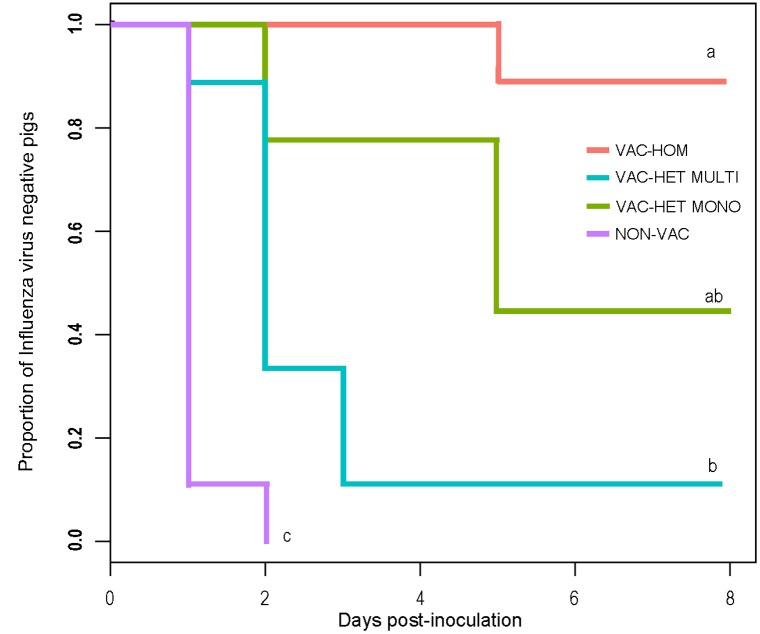
Survival curve of influenza A virus pig infection comparing different vaccination treatments. Comparison of pigs that were immunized with different vaccines based on RRT-PCR: VAC-HOM: homologous vaccine; VAC-HET MULTI: heterologous multivalent vaccine; VAC-HET MONO: heterologous monovalent vaccine; NON-VAC: non-vaccinated. Letters above the curves show differences statistically significant between groups at 95% confidence level.

The proportion of pigs infected was significantly higher in the NON-VAC group than the vaccinated ones (p < 0.05) per the Kaplan-Meier survival analysis. Also, the percentage of infected pigs in VAC-HOM group was significantly lower than VAC-HET MULTI, but not the VAC-HET MONO, which 95% confidence intervals overlap (Fig 1).

**Fig 2:**
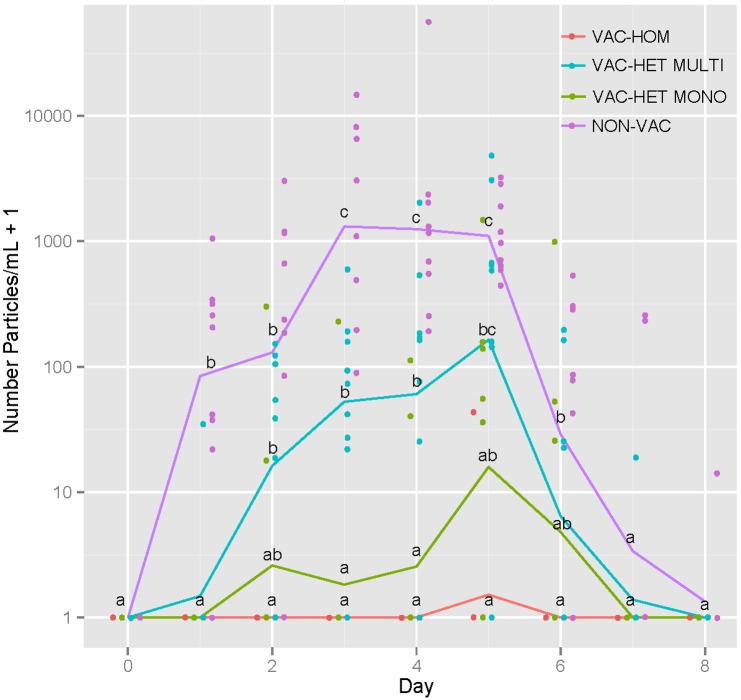
Nasal shedding of influenza A virus (IAV). Scatter plot of nasal swab IAV shedding based on RRT-PCR results comparing different groups: VAC-HOM: homologous vaccine; VAC-HET MULTI: heterologous multivalent vaccine; VAC-HET MONO: heterologous monovalent vaccine; NON-VAC: non-vaccinated. Lines are connecting the means. Different letters indicate statistical differences between groups evaluated by day (p < 0.05).

Viral quantification was expressed as average TCID_50_ equivalents (equi)/ml (Table 3). The viral load in nasal swabs ranged from 1.1 to 3.9 TCID_50_ equi/ml. Pigs in the NON-VAC group had higher amounts of nasal virus shedding most of the sampling days compared to vaccinated groups (p<0.05). Additionally, the VAC-HET MULTI group had higher levels of IAV compared with VAC-HET MONO and VAC-HOM groups (Fig 2). Oral fluid results were in agreement with nasal swab results although statistics were not performed due to the low number of observations.

All air samples in the vaccinated groups tested negative by RRT-PCR. Air samples collected at days 1, 2 and 3 from NON-VAC pigs tested positive by RRT-PCR but negative by virus isolation after one passage in MDCK cells. Overall, the levels of virus detection ranged between 1 to 1.6 log TCID_50_ equi/ml. (Table 1, Fig 2).

## Discussion

Understanding the transmission of IAV across pig populations and species is important to minimize the risk of generation of new viruses with pandemic potential. Airborne transmission plays a role in the spread of IAV in pig populations [18] and vaccination has been shown to reduce influenza transmission [27,28]; however, data related to the potential decrease of biosaerosol generation by vaccines is scarce. In this study, both the decrease in IAV shedding and the increase in environmental temperature may have contributed to the likelihood of bioaerosol detection in vaccinated animals.

To emulate field conditions, the treatment groups formed in this study were comprised of pigs with varying degrees of cross-protective immunity, as it likely occurs when pigs obtain maternally derived antibodies or are challenged throughout their lifespan. Lower serologic cross-reactivity has been associated with decreased protection in previous studies [26] and this agrees with what was seen herein where pigs from heterologus vaccination groups were actively shedding at different levels.

In our study overall detection of IAV in aerosols was lower than previously reported [17,40]. In this study, we were not able to detect IAV in air samples from vaccinated groups even when pigs were actively shedding virus. One factor that may have contributed to this is that pigs in vaccinated groups had lower nasal shedding compared to that of non-vaccinated animals (Fig 2). An explanation for the lower detection levels observed in the non-vaccinated pigs could be due to the study environmental conditions. The present study was carried out during an unexpected heatwave, with an average room temperature of 27°C (90% of readings ±1.5°C), in contrast to lower temperatures reported in previous studies [15,40]. Higher temperatures have been associated with lower aerosol transmission or even cessation of transmission at temperatures above 30°C [31]. Lower temperatures may allow higher virus survival and increase viral spread from the lungs compared with higher temperatures which inactivate viral particles [30]. Relative humidity conditions may have also played a role in the survival of the virus although RH% effect is considered less important than that of temperature, especially when temperature is high [31]. In our study, RH% remained constant at 53% (95% of readings ±7.8%) and is considered to have had a lesser role in virus survivability compared to temperature. Airborne transmission at a high temperature of 30°C has been documented when relative humidity is 20% [32]. Nevertheless, in our study we were able to detect IAV in the air even under warm conditions albeit at low frequency, which may indicate that aerosol transmission can still play a role in transmission of influenza in warm climates, particularly when individuals are in close contact.

Whether a similar reduction would be seen under cold weather conditions needs to be further investigated. Lowen and Palese have suggested that transmission of IAV in warm climates is more contact related whereas transmission in temperate conditions is predominantly by aerosol [32].

In this study, we focused our efforts on detecting IAV directly from air samples instead of evaluating transmission between pigs. Advantages of doing so include the ability to detect IAV directly from the air to evaluate feasibility of sampling methods to assess environmental virus spread. Detection of IAV from air samples using the air collection equipment and methods described here have been used before in both experimental and field based studies. In these other studies, IAV was detected and isolated from air samples from naïve and immune pigs experimentally infected and from the interior and exterior of pig farms [15,16,19,40]. Therefore we speculate that the lack of virus detection in the air in the vaccinated groups is the result of reduced shedding by vaccinated animals in combination with decreased stability of the virus in the warm environmental conditions of this study rather than an effect of the air sampling methods.

Our results are in contrast with a previous report where IAV was detected in aerosols from vaccinated animals [15,17] and indirect transmission was reported between vaccinated and sentinel pigs [41]. Whether the differences between studies are due to differences in shedding levels, cross-reactivity between the vaccine and the challenge virus or environmental conditions supportive of virus inactivation is unclear. The authors speculate that both the decrease in shedding in vaccinated animals and the increase in environmental temperature conditions were responsible for the inability to detect virus in the air and that may account for the differences between the studies. Nevertheless, the authors also speculate that vaccination even when shedding is not fully prevented, may still aid in decreasing population shedding therefore vaccination should be considered as part of a comprehensive influenza control program.

## Conclusions

In conclusion, our results suggest that both the decrease in shedding and the increase in environmental temperature may contribute to the reduction in airborne IAV detection in vaccinated pigs. However, further studies are needed to better assess the effect of vaccination and environmental conditions on IAV aerosol dissemination in particular during cold weather conditions when IAV infections are considered more clinically relevant.

## Supporting information

S1 Table File containing individual-level raw data of ELISA, HI, RRT-PCR, room temperature and relative humidity results. (XLSX)

S1 Fig Representation of percentiles of weather conditions. Y axis indicate percentage of readings and X axis indicate relative humidity or temperature. Ninety five percent of the readings were 53% ±7.8 RH and 27 °C ±11.6.
(TIFF)
