# Supplementary figures and images for: Detection of influenza A virus in aerosols of vaccinated and non-vaccinated pigs in a warm environment
Source: PLoS One. 2018 May 21;13(5):e0197600. doi: 10.1371/journal.pone.0197600 (PMC5962048; doi:10.1371/journal.pone.0197600)

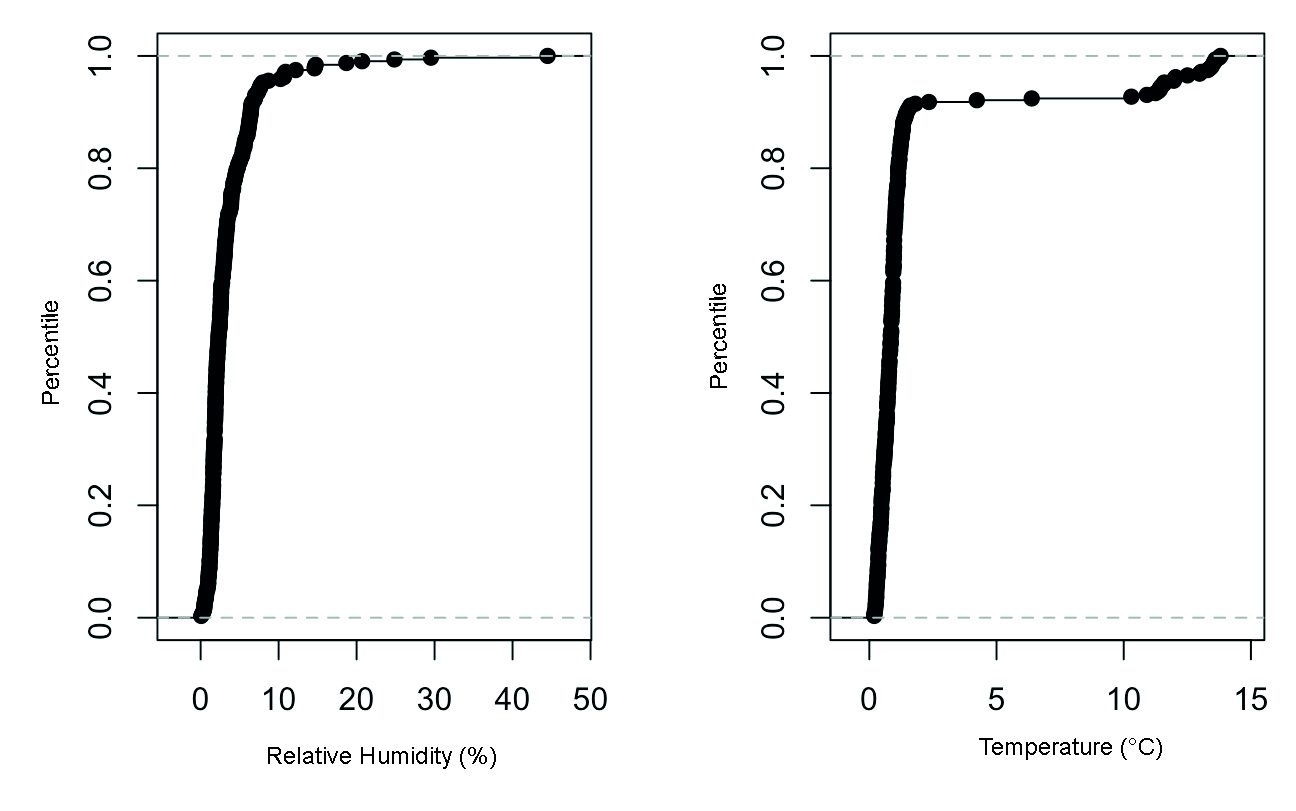

Supplement: S1 Fig — Y axis indicate percentage of readings and X axis indicate relative humidity or temperature. Ninety five percent of the readings were 53% ±7.8 RH and 27 °C ±11.6. (TIFF) [file pone.0197600.s002.tiff]
